# Reeler Domain-Containing Proteins Involved in the Antibacterial Immunity of Shrimp *Litopenaeus vannamei*

**DOI:** 10.3390/md23050215

**Published:** 2025-05-20

**Authors:** Jianying Qi, Guoqing Dai, Huiling Xing, Zhibin Fu, Sheng Ke, Lili Shi

**Affiliations:** 1 College of Fisheries, Guangdong Ocean University, Zhanjiang 524088, China; 13435076501@163.com (J.Q.);; 2 Shenzhen Institute of Guangdong Ocean University, Shenzhen 518114, China

**Keywords:** *Litopenaeus vannamei*, Reeler, antibacterial immunity, RNA interference, recombinant protein

## Abstract

Like other invertebrates, *Litopenaeus vannamei* lacks adaptive immunity and relies mainly on innate immunity for defense against foreign pathogens. In this study, three distinct Reeler domain-containing molecules were discovered in *L. vannamei*, designated as *LvReeler1*, *LvReeler2*, and *LvReeler3*. Analysis of tissue-specific expression patterns indicated that *LvReeler1* showed predominant expression in the stomach, whereas *LvReeler2* and *LvReeler3* demonstrated peak transcriptional activity within gill tissues. The expression of these molecules was induced by *Vibrio parahaemolyticus*. In vivo interference with *LvReelers* expressions via dsRNA significantly increased the mortality rate of *L. vannamei*, while also leading to a marked increase in the bacterial load of *V. parahaemolyticus* in the gills. Additionally, recombinant proteins of *LvReeler1* (rLvReeler1), *LvReeler2* (rLvReeler2), and *LvReeler3* (rLvReeler3) were successfully expressed in *Escherichia coli*. Antibacterial assays demonstrated that rLvReelers inhibited the growth of *V. parahaemolyticus*, *Vibrio alginolyticus*, and *Vibrio harveyi*, with rLvReeler3 exhibiting the strongest inhibitory activity. Scanning electron microscopy (SEM) observations revealed that rLvReeler3 caused bacterial aggregates to disintegrate after binding to *V. parahaemolyticus* and *V. alginolyticus*. In conclusion, *LvReelers* play an active role in the antimicrobial immune response of *L. vannamei.*

## 1. Introduction

*Litopenaeus vannamei* (colloquially termed the Pacific white shrimp or South American white shrimp) dominates global aquaculture as the primary cultivated species, accounting for more than 80% of total shrimp production worldwide [1]. In recent years, the prevalence of shrimp diseases, particularly bacterial infections caused by *Vibrio* species, has risen sharply, leading to high mortality rates and considerable economic losses, thereby threatening the sustainability of global shrimp farming [2,3]. As an invertebrate, *L. vannamei* depends on its innate immune system, which encompasses both humoral and cellular immunity, to defend against infections [4]. Humoral immunity is mediated through the synthesis and secretion of immune proteins, such as antimicrobial peptides, while cellular immunity involves the activation of phagocytic cells, encapsulation, and nodulation [5]. Together, these immune responses constitute the defense against bacterial, fungal, and viral infections in insects, crustaceans, and other invertebrates. Therefore, understanding the immune mechanisms of shrimp is essential for disease control and prevention [6,7].

The Reeler domain was first characterized in Reelin, an extracellular matrix protein identified in murine systems [8], positioned within its N-terminal segment [9]. This 388 kDa glycoprotein serves critical functions during mammalian central nervous system development [10], modulating neuronal migration and spatial organization [11,12]. Insect model studies indicate that Reeler domain-containing molecules potentially participate in immunological processes. A pioneering discovery in arthropods revealed a 19 kDa hemolymph protein from *Locusta migratoria* as the inaugural insect-derived Reeler domain carrier, primarily localized within adult specimens’ circulatory fluid, with expression being upregulated through juvenile hormonal regulation [13]. In the Indian oak silkworm, a novel protein was identified that participates in the nodulation response of cellular immunity, and it was consequently named Noduler. Noduler is a 168-amino-acid protein, with the Reeler domain spanning nearly the entire protein, forming nodules through a melanization cascade reaction to contribute to immune defense [14]. Functioning as a critical element in nodulation processes, this protein mediates adhesion to microbial pathogens (bacteria and yeast) and arthropod hemocytes. Its specific binding capacity toward bacterial cell wall components initiates melanin deposition cascades, facilitating immune encapsulation and displaying hallmarks of insect pattern recognition receptors (PRRs). Orthologous Noduler genes have been characterized across multiple insect taxa, including *Hyphantria cunea* [15], *Manduca sexta* [16], *Lonomia obliqua* [17], and *Bombyx mori* [18]. Pathogen-challenged *B. mori* larvae with Reeler1 gene silencing exhibit diminished nodule counts, a phenotypic defect reversible through supplementation with recombinant Reeler1. These observations imply that *Bombyx mori* Reeler1 contributes mechanistically to nodule biogenesis and serves as an indispensable mediator within melanization pathways.

Within crustacean species, a Reeler domain-containing protein designated PcReeler was initially characterized in *Procambarus clarkii*. This molecule exhibits predominant expression within gills, with transcriptional activation occurring following microbial challenge. Such tissue-specific immune gene expression is evolutionarily conserved in arthropods. In *B. mori*, Reeler homolog BmReeler1 exhibits hemocyte- and fat body-enriched expression that correlates with the immune functions of these tissues [18]. This spatial regulation likely reflects the functional specialization of immune defense mechanisms. Mortality rates in crayfish substantially increased when *PcReeler* expression was suppressed through RNA interference techniques. Experimental evidence confirms that recombinant PcReeler demonstrates binding affinity toward microbial polysaccharides and whole bacterial cells, effectively suppressing biofilm development [19]. Transcriptome profiling of *Vibrio parahaemolyticus*-infected *L. vannamei* further identified multiple Reeler domain-bearing proteins displaying marked transcriptional upregulation relative to uninfected controls, implying functional participation in anti-*Vibrio* defense mechanisms. In this study, three proteins containing the Reeler domain were cloned and identified, and their roles in antibacterial immunity were confirmed through RNA interference (RNAi) and recombinant protein assays. Similar functions have also been identified in *P. clarkii*, where Reeler domain-containing proteins such as PcReeler contribute to antibacterial immunity [19].

## 2. Results

### 2.1. Cloning and Sequence Analysis of LvReeler

**Figure A1:**
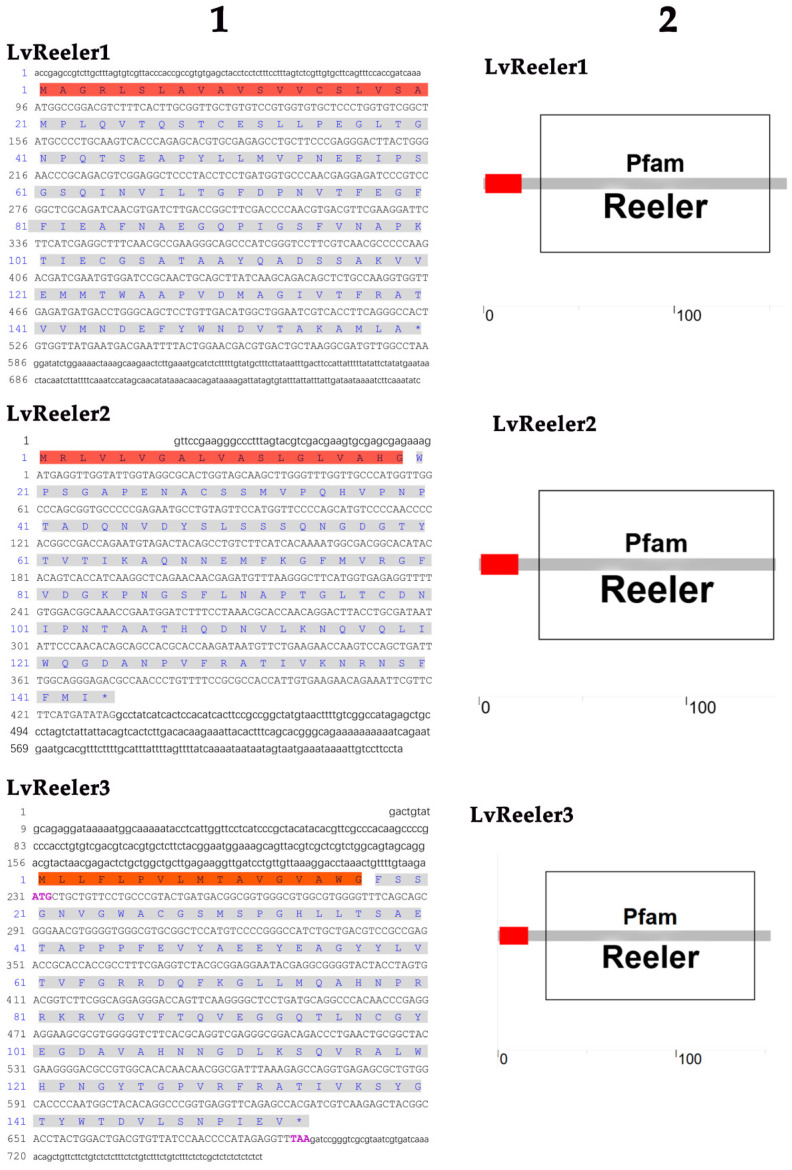
Sequence characterization of LvReeler proteins. (**1**) Molecular sequences from *L. vannamei* encoding *LvReeler*, presenting both nucleotide sequences and their corresponding deduced amino acid residues. The signal peptide is depicted with red highlighting, with initiation/termination codons marked in purple lettering. The corresponding deduced amino acid sequence is displayed above the cDNA sequence. (**2**) Domain architecture schematic of *LvReeler.* Conserved structural domains were computationally predicted via the SMART algorithm.

The open reading frames (ORFs) (Figure A1(1)) of *LvReeler1* (Genbank No. XM_027352375.2), *LvReeler2* (Genbank No. XM_027373871.2), and *LvReeler3* (Genbank No. XM_027352575.2) are 480 bp, 432 bp, and 462 bp in length, encoding 159, 143, and 153 amino acids, respectively. The molecular weights of *LvReeler1*, *LvReeler2*, and *LvReeler3* proteins are 16,821.23 Da, 15,364.36 Da, and 16,818.10 Da, respectively, with theoretical isoelectric points (pI) of 4.00, 6.81, and 6.90. Similarity analysis revealed that the amino acid sequences of the three LvReeler proteins share 35% to 45% homology. SMART analysis revealed that all three LvReeler proteins possess both a conserved Reeler domain (gray, Figure A1(2)) and an N-terminal signal peptide (red, Figure A1(2)). The Reeler domain, spanning nearly the complete length of each protein, displays structural characteristics consistent with its putative role in immune recognition and microbial binding. Each protein contains a 15–30 amino-acid signal peptides, strongly suggesting extracellular secretion and supporting their potential involvement in humoral immunity.

### 2.2. Multiple-Sequence Aligment and Phylogenetic Analysis

**Figure 1:**
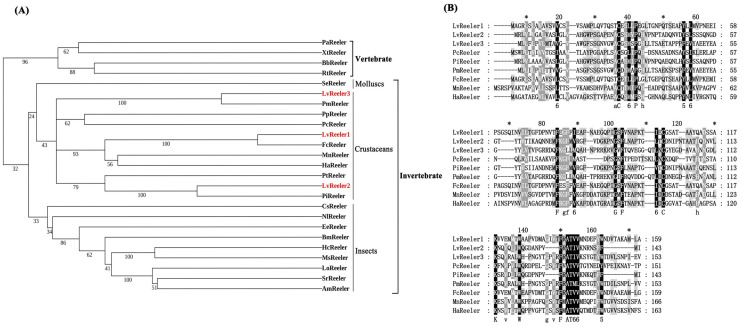
(**A**) Phylogenetic tree analysis using the neighbor-joining method. A rooted tree was constructed using MEGA 6.0 software (http://www.megasoftware.net/index.html, accessed on 15 November 2024) with 1000 bootstrap replicates. *LvReelers* are marked in red. Abbreviations are as follows: PaReeler, *Protopterus annectens* (XP_043942745.1); XtReeler, *Xenopus tropicalis* (XP_012817658.1); BbReeler, *Bufo bufo* (XP_040263400.1); RtReeler, *Rana temporaria* (XP_040216121.1); SeReeler, *Saccostrea echinata* (XP_061191452.1); PmReeler, *Penaeus monodon* (XP_037796316.1); PpReeler, *Pollicipes pollicipes*(XP_037085477.1); PcReeler, *Procambarus clarkii* (XP_045622215.1); FcReeler, *Penaeus chinensis* (XP_047480392.1); MnReeler, *Macrobrachium nipponense* (XP_064113092.1); HaReeler, *Homarus americanus* (XP_042214841.1); PtReeler, *Portunus trituberculatus* (XP_045137636.1); PiReeler, *Penaeus indicus* (XP_063609294.1); CsReeler, *Cryptotermes secundus* (XP_023706682.1); NlReeler, *Nilaparvata lugens* (AGK40918.1); EeReeler, *Euphydryas editha* (CAH2104874.1); BmReeler, *Bombyx mori* (ADZ40416.1); HcReeler, *Hyphantria cunea* (AAD09280.1); MsReeler, *Manduca sexta* (AAO21507.1); LoReeler, *Lonomia obliqua* (AAV91350.1); SrReeler, *Samia ricini* (BAD05929.1); AmReeler, *Antheraea mylitta* (ABG72705.1). (**B**) Multiple-sequence alignment of the deduced amino acid sequences. The asterisk (*) is used as a spacing marker to help quickly locate specific amino acid positions, with an asterisk placed every 10 amino acids. Dark-shaded regions represent fully conserved residues across all sequences, with gray shading indicating conservative substitutions. Gaps introduced as dashes maintain alignment integrity. Accession numbers for alignment sequences match those listed in (**A**).

Phylogenetic analysis employing the neighbor-joining (NJ) method categorizes Reeler proteins into two evolutionarily divergent clades: invertebrate and vertebrate lineages. *LvReeler* clusters within the invertebrate clade, exhibiting close phylogenetic affinity with Reeler homologs from diverse insect species, notably *Cryptotermes secundus*, *Nilaparvata lugens*, *Euphydryas editha*, *Bombyx mori*, *Hyphantria cunea*, *Manduca sexta*, *L. obliqua*, *Samia ricini*, and *Antheraea mylitta* (Figure 1A). Multiple-sequence alignment (Figure 1B) indicates that *LvReeler* shares high similarity with Reeler proteins from other shrimp species, with similarity ranging from 30% to 92%. *LvReeler1*, *LvReeler2*, and *LvReeler3* show the highest similarity to those from *Penaeus chinensis* (91.19%), *Penaeus indicus* (91.61%), and *Penaeus monodon* (88.24%), respectively.

### 2.3. Tissue Distribution of LvReeler in Healthy L. vannamei

**Figure 2:**
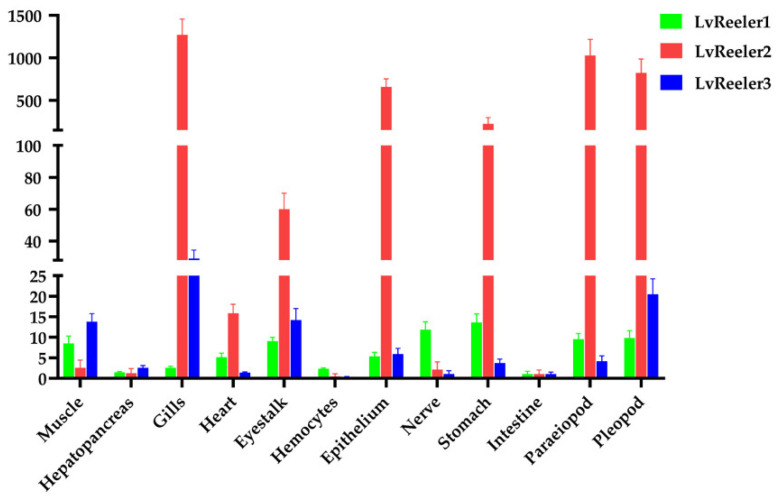
Tissue distribution of *LvReelers* expression in healthy *L. vannamei*. Tissue samples were collected from 10 healthy specimens. Transcript abundance of all *LvReeler* genes were normalized to their expression levels in intestinal tissue (set as 1.0-fold). cDNA templates employed in qPCR assays underwent normalization against the endogenous control *LvEF-1α*. Data were derived from triplicate biological replicates and presented as mean values ± SD deviation.

The expression profiles of *LvReelers* in various tissues were detected by qRT-PCR, revealing the presence of *LvReelers* transcripts in all tested tissues (Figure 2). Specifically, *LvReeler1* showed higher expression in the stomach, muscle, and eyestalk, with the highest expression in the stomach, approximately 13.6 times greater than the baseline level (1.0). *LvReeler2* and *LvReeler3* exhibited the highest expression in the gills, with expression levels 1270.4 and 29.1 times higher than the baseline, respectively. Additionally, *LvReeler2* and *LvReeler3* also expressed at varying levels in other tissues, with relatively high expression observed in the pleopod, paraeiopod, epithelium, stomach, and eyestalk. This tissue-specific expression pattern suggests that different LvReeler proteins may have specialized roles and cooperate in shrimp immune defense, collectively forming the immune defense network.

### 2.4. Expression Profiles of LvReeler in Response to V. parahaemolyticus Infection

**Figure 3:**
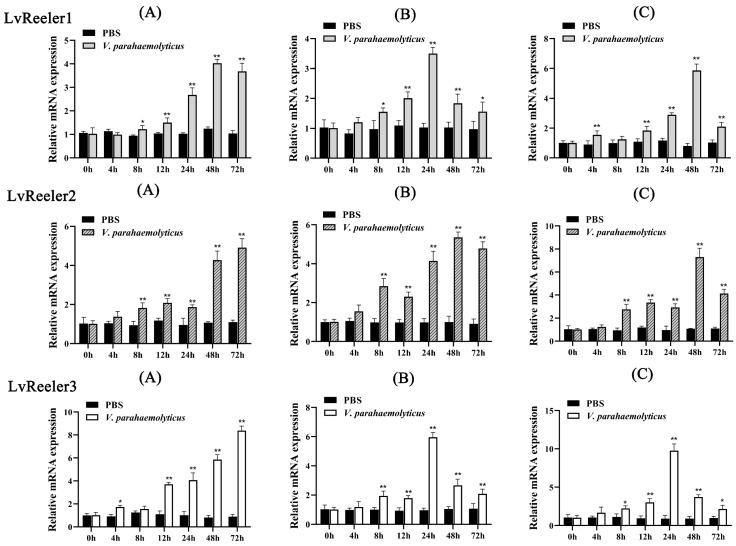
Expression profiles of *LvReelers* in immune-challenged *L. vannamei*. Relative expression levels of *LvReelers* were quantified across hemocytes (**A**), hepatopancreas (**B**), and gills (**C**) following *V. parahaemolyticus* exposure. Transcript abundance was normalized against *LvEF-1α* using the Livak (2^−ΔΔCt^) method, with values derived from three biological replicates (mean ± SD). Statistical analyses employed Student’s *t*-test (* *p* < 0.05, ** *p* < 0.01 versus unchallenged controls).

Major immune-responsive tissues in *L. vannamei* (hemocytes, hepatopancreas, and gills) were chosen to investigate temporal expression dynamics of *LvReeler* genes following *V. parahaemolyticus* challenge. As illustrated in Figure 3, pathogen exposure induced differential upregulation patterns of *LvReeler* transcripts across examined tissues. In hemocytic populations (Figure 3A), *LvReeler1* demonstrated maximal induction at 48 h post-infection (3.55-fold relative to uninfected controls), while *LvReeler2* and *LvReeler3* attained peak expression at 72 h (4.85- and 9.37-fold increases versus controls, respectively). Hepatopancreas analysis (Figure 3B) revealed biphasic regulation of *LvReeler* genes, characterized by initial upregulation followed by progressive decline. *LvReeler1* and *LvReeler3* exhibited maximal transcript levels at 24 h (3.4- and 6.18-fold inductions, respectively), whereas *LvReeler2* showed highest expression at 48 h (5.33-fold elevation compared to baseline). In the gills (Figure 3C), the expression of *LvReeler1* and *LvReeler2* gradually increased from 4 to 48 h, reaching their highest levels, 7.3 and 6.84 times higher than the control group, respectively; *LvReeler3* peaked at 24 h, 10.85 times higher than the control group, and decreased to 2.2 times the control level at 72 h. These results demonstrate that *V. parahaemolyticus* infection has a varying impact on the expressions of *LvReeler* genes, with different expression patterns observed across tissues. This may be due to differences in immune response mechanisms and cellular composition across tissues, leading to varying speeds and modes of response to pathogen invasion.

### 2.5. Function of LvReelers in V. parahaemolyticus Infection

**Figure 4:**
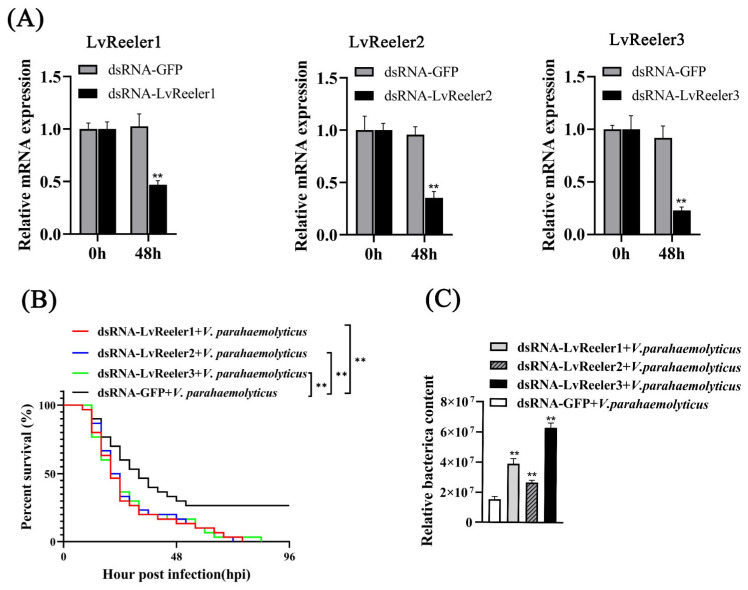
Functional characterization of *LvReelers* during *V. parahaemolyticus* infection. (**A**) RNAi-mediated knockdown efficacy for *LvReelers* was quantified via qRT-PCR using *EF-1α* for normalization. Gill tissues sampling occurred 48 h post-dsRNA administration. (**B**) Survival dynamics of *V. parahaemolyticus*-challenged shrimp. Dual independent trials yielded consistent mortality patterns. Inter-group survival differences were analyzed via the log-rank χ^2^ test. (**C**) Pathogen load in gill tissues of dsRNA-*LvReeler*-treated *L. vannamei* was assessed through qPCR at 24 h post-infection, following established methodologies [20]. Student’s *t*-test determined statistical significance (** *p* < 0.01 versus untreated controls).

RNA interference-mediated knockdown efficacy for *LvReelers* was assessed via qRT-PCR. Transcript levels of *LvReelers* in RNAi-treated cohorts exhibited significant suppression relative to dsRNA-GFP controls, as depicted in Figure 4A, demonstrating respective decreases of 46.97%, 42.25%, and 32.91%. Injections of dsRNA-GFP in the control groups did not suppress the expressions of *LvReelers* and did not significantly induce downregulation (*p* > 0.05).

To elucidate the functional contributions of *LvReelers* to anti-*V. parahaemolyticus* defense mechanisms, post-challenge survival kinetics in dsRNA-administered *L. vannamei* were systematically monitored. Compared to the control groups (Figure 4B), the experimental groups with silenced *LvReelers* exhibited significantly lower survival rates. In the dsRNA-silenced experimental groups, the survival rate of shrimp was 0%, while in the control groups, the survival rate was 26.67%. Quantitative analysis of gills bacterial colonization in *L. vannamei* was conducted at 24 h post-treatment. RNAi-treated cohorts demonstrated markedly elevated microbial counts relative to unmodified controls (Figure 4C), with the most pronounced enhancement observed in the dsRNA-LvReeler3 co-challenged cohort. These findings establish *LvReeler* genes as essential mediators of anti-*V. parahaemolyticus* defense mechanisms in *L. vannamei*.

### 2.6. Recombinant Expression of LvReelers

**Figure 5:**
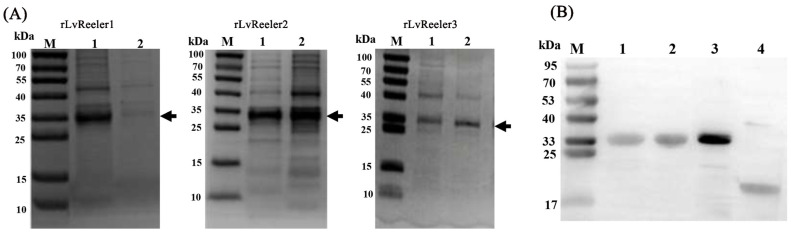
Heterologous expression and immunoblot analysis of *LvReelers*. (**A**) Electrophoretic separation via SDS-PAGE of Trx-fused *LvReelers* heterologously expressed in *E. coli*. Sample preparation involved mixing with equivalent volumes of loading buffer, thermal denaturation at 100 °C for 5 min, and subsequent electrophoretic resolution. Post-separation, gels were visualized using Coomassie Brilliant Blue R-250 staining followed by destaining. Lane designations: M, molecular mass standards; Lane 1, soluble cellular lysate; Lane 2, insoluble cellular fraction. (**B**) Western blot detection using anti-His antibody. Lane 1: purified *LvReeler1*; Lane 2: purified *LvReeler2*; Lane 3: purified *LvReeler3*; Lane 4: purified Trx control.

Heterologous expression of recombinant rLvReelers was achieved in *E. coli*, with soluble expression confirmed in cellular fractions (Figure 5A). Induction with 1 mM IPTG at 20 °C for 6 h facilitated recombinant protein production, followed by bacterial pellet collection via centrifugation. Affinity-purified proteins obtained through Ni-NTA agarose chromatography were resolved on 15% SDS-PAGE, displaying discrete electrophoretic bands corresponding to each recombinant protein. The calculated molecular masses of rLvReeler1 (34.9 kDa), rLvReeler2 (33.7 kDa), and rLvReeler3 (35.0 kDa) accounted for both the native proteins (15.7 kDa, 14.5 kDa, and 15.8 kDa, respectively) and the 19.2 kDa Trx-His tag. This alignment between theoretical predictions and electrophoretic mobility confirmed successful expression of structurally intact fusion proteins. Control experiments utilized a rTrx protein purified from an empty pET-32a vector. Immunoblotting validated immunoreactive bands for rLvReeler1, rLvReeler2, rLvReeler3, and rTrx, exhibiting electrophoretic mobilities concordant with predicted molecular masses (Figure 5B). These outcomes establish essential molecular tools and foundational references for functional exploration and applied research on LvReeler proteins.

### 2.7. Antibacterial Activity of rLvReelers In Vitro

The heterologously expressed rLvReelers demonstrated differential antimicrobial efficacy against *Vibrio* species. As quantified in Table 1, rLvReeler1 displayed minimum inhibitory concentration (MIC) values of 60 μM toward both *V. parahaemolyticus* and *V. harveyi*, while showing enhanced potency against *V. alginolyticus* (MIC = 50 μM). rLvReeler2 exhibited reduced inhibitory capacity against *V. alginolyticus* (MIC = 60 μM) and *V. harveyi* (MIC = 50 μM) but showed maximal suppression of *V. parahaemolyticus* (MIC = 40 μM). Notably, rLvReeler3 achieved the lowest MIC values among tested variants, with 30 μM against *V. parahaemolyticus* and *V. alginolyticus*, alongside moderate inhibition of *V. harveyi* (MIC = 50 μM). These findings establish rLvReeler3 as the most potent inhibitor of *V. parahaemolyticus* and *V. alginolyticus* among the recombinant proteins. The differences in antibacterial activity may be attributed to the structural characteristics of the proteins and their affinity for binding sites on the bacterial surface.

### 2.8. Morphological Changes in Vibrio Using Scanning Electron Microscopy (SEM)

**Figure 6:**
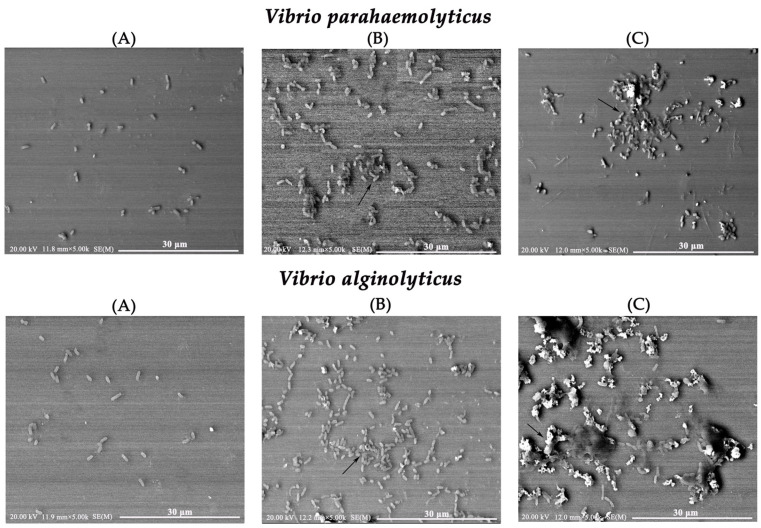
SEM analysis of morphological changes in *V. parahaemolyticus* and *V. alginolyticus* after treatment with rLvReeler3 from *L. vannamei*. (**A**) Control group treated with rTrx-His-Tag; (**B**) bacterial morphology after treatment with 80 μM rLvReeler3 for 1 h, with arrows indicating bacterial aggregation; (**C**) bacterial morphology after treatment with 80 μM rLvReeler3 for 2 h, with arrows indicating the disintegration of aggregates.

To further characterize the antibacterial activity of rLvReeler3, SEM was used to observe the morphological changes in *V. parahaemolyticus* and *V. alginolyticus* following treatment with rLvReeler3 (Figure 6). Untreated *V. parahaemolyticus* and *V. alginolyticus* appeared dispersed or in small aggregates, with intact structures and well-defined morphology (Figure 6A). After treatment with rLvReeler3, within 1 h, the bacteria aggregated into irregular clusters, with some bacteria forming clumps (Figure 6B). After 2 h of incubation, bacterial aggregation was suppressed, small aggregates began to disintegrate, and a diffuse high-density area appeared around the bacteria (Figure 6C). Based on these morphological changes, it is hypothesized that rLvReeler3 may induce aggregation of the cell membranes of *V. parahaemolyticus* and *V. alginolyticus*, thereby disrupting their normal physiological functions and exerting antibacterial effects.

## 3. Discussion

Within arthropod immune defense mechanisms, Reeler domain-containing proteins have garnered significant attention as critical research subjects in immunology. Notably in taxa, including insects and *P. clarkii*, these proteins mediate essential immunological mechanisms such as nodule biogenesis, melanization cascades, and microbial community homeostasis regulation [19,21,22]. For example, the Noduler protein in insects stimulates cell proliferation by activating the p38/MAPK signaling pathway and participates in nodule formation, a process that begins with the aggregation of hemocytes and culminates in the formation of dark nodules through melanization [23,24]. Functioning as an insect-derived PRR, the Noduler protein demonstrates binding affinity toward microbial components, including bacteria and yeast, as well as host hemocytes, with particular specificity for bacterial cell wall constituents, facilitating the initiation of nodulation processes [14,25,26]. Typically, the nodulation process requires the interaction of recognition molecules with hemocytes and invading microorganisms [27,28,29]. It acts as an upstream factor in the phenoloxidase cascade, and gene knockout does not directly affect the Toll/Imd pathway [30]. Given the conservation of immune mechanisms in arthropods, it is speculated that similar functional proteins may also exist in *L. vannamei*.

Based on the transcriptomic data from *L. vannamei* infected with *V. parahaemolyticus*, this study successfully identified three proteins containing a single Reeler domain—*LvReeler1*, *LvReeler2*, and *LvReeler3*. Additionally, multiple homologs of proteins containing the Reeler domain have been identified in other species, such as *Anopheles gambiae*, *Drosophila melanogaster*, *A. mylitta*, *Aedes aegypti*, and *Tribolium castaneum* [14,31,32]. Gene amplification of these homologs likely provides a crucial foundation for the diversification of pathogen defense mechanisms, enabling organisms to deploy a broader range of strategies when confronted with diverse pathogen threats. Similar to insect nodule proteins, the three LvReeler proteins in *L. vannamei* consist of a signal peptide and a single Reeler domain that spans nearly the entire length of the protein. In terms of tissue distribution, different *LvReeler* members exhibit distinct expression patterns, suggesting a close association with their functional specialization in shrimp immune defense. *LvReeler2* and *LvReeler3* are highly expressed in the gills, which are critical immune defense organs in crustaceans [33], indicating their significant roles in gill immunity. In contrast, *LvReeler1* is highly expressed in tissues such as the stomach, potentially playing a role in the immune defense of the digestive system. This finding enhances our understanding of immune specialization across different tissues in shrimp.

*V. parahaemolyticus*, *V. alginolyticus*, and *V. harveyi* have been identified as major pathogenic bacteria in shrimp [34]. Upon *V. parahaemolyticus* invasion, the expression of *LvReelers* in the gills is rapidly upregulated, highlighting their role in the immune response. A similar expression pattern has been observed in *P. clarkii* with *PcReeler*. To explore their role in innate immunity, we investigated the function of *LvReelers* using RNAi technology. RNAi-mediated silencing of *LvReelers* suppressed immune responses in shrimp infected with *V. parahaemolyticus*. Mortality assays revealed that shrimp with silenced *LvReelers* exhibited significantly higher mortality rates compared to the control group prior to bacterial infection, and bacterial loads in the gills were also markedly increased. The individual knockdown experiments demonstrated the essential role of each LvReeler protein in antibacterial defense, yet their functional interrelationships remain to be elucidated. Future investigations employing combinatorial RNA interference targeting multiple *LvReelers* would provide critical insights into their cooperative mechanisms in shrimp immunity. These findings confirm the critical role of *LvReelers* in antibacterial immunity and suggest their potential as key regulatory nodes in the shrimp immune defense system.

The potential broad-spectrum activity of LvReeler proteins warrants investigation, given the conserved nature of arthropod immunity. Structural and functional parallels with Reeler-domain proteins in other species (e.g., insect Noduler proteins that recognize diverse pathogens) suggest *LvReelers* may also respond to non-*Vibrio* microorganisms. Their upregulated expression during bacterial challenge and antimicrobial activity indicate possible roles against Gram-positive bacteria, fungi, or viruses, though the exact specificity requires validation through systematic challenge experiments with varied pathogens and subsequent immune response monitoring. Furthermore, in arthropods, proteins containing the Reeler domain have been demonstrated to participate in antibacterial immune responses. For instance, the knockdown of PcReeler in *P. clarkii* leads to increased bacterial abundance in the gills and disrupts microbial community stability [19]. In *A. mylitta*, the knockdown of the *Noduler* gene results in a significant increase in bacterial load and a marked reduction in nodule formation [14]. Similarly, BmReeler1 in *B. mori* is strongly induced upon bacterial infection, and double-stranded RNAi reduces nodule formation in larvae [18]. These studies collectively underscore the significant role of Reeler domain-containing proteins in the immune processes of arthropods.

To further investigate the antibacterial activity of *LvReelers*, we successfully expressed recombinant proteins rLvReelers in *Escherichia coli* Origami (DE3). Quantitative analyses revealed measurable antimicrobial efficacy of rLvReelers against *V. parahaemolyticus*, *V. alginolyticus*, and *V. harveyi*, displaying MICs spanning 30–60 μM. Notably, rLvReeler3 exhibited potent growth suppression against *V. parahaemolyticus* and *V. alginolyticus*, with equivalent MICs of 30 μM for both pathogens. SEM observations demonstrated rLvReeler3’s capacity to disrupt bacterial aggregation structures in both *V. parahaemolyticus* and *V. alginolyticus*. While SEM revealed that rLvReeler3 induced disruption of bacterial aggregation, its exact antibacterial mechanism requires further investigation. Future studies should employ membrane permeability assays (e.g., propidium iodide uptake) to determine whether rLvReelers directly damage bacterial membranes. Entomological studies confirm Reeler-mediated nodulation through microbial and hemocyte binding coupled with melanization-based bactericidal mechanisms [18,35]. In *P. clarkii*, PcReeler demonstrates dual functionality by impeding bacterial proliferation and biofilm development [19]. The observed *Vibrio*-inhibitory properties of rLvReelers in *L. vannamei* highlight their potential as biocontrol candidates, providing innovative strategies for shrimp vibriosis management. However, several questions remain to be explored, such as whether LvReeler proteins induce *Vibrio* cell membrane rupture and whether they bind to hemocytes to form nodules. Further investigation of these issues will help to comprehensively elucidate the molecular mechanisms underlying their antibacterial immunity.

Although in vitro experiments indicate that recombinant LvReeler protein exhibits antibacterial activity against Vibrio species, these experiments were conducted under highly concentrated and purified conditions, and the function of the protein in the in vivo environment remains unclear. It is currently unknown whether the protein exerts its effect by directly killing bacteria, promoting bacterial aggregation, enhancing phagocytosis, or participating in other immune processes. While RNAi experiments have confirmed the crucial role of the *LvReeler* in antibacterial immunity, there is a lack of evidence linking changes in gene expression with specific steps of antibacterial function. Future research should aim to bridge this knowledge gap by conducting in-depth histological and immunological studies and signaling pathway analysis to further elucidate the in vivo role and mechanisms of LvReeler protein.

## 4. Materials and Methods

### 4.1. Animals and Microorganisms

Specimens of *L. vannamei* (6–8 g) were sourced from an aquaculture facility in Zhanjiang, Guangdong Province, China. Following a 7-day acclimatization period in a recirculating aquaculture system (RAS), shrimp were maintained under controlled parameters: salinity 27‰, temperature 25–27 °C. A commercial pellet diet was administered twice per day. Experimental *Vibrio* strains (*V. parahaemolyticus* (ATCC17802), *V. alginolyticus* (ATCC17749) and *V. harveyi* (ATCC35084)), utilized in this study originated from our laboratory stock cultures.

### 4.2. Cloning of the ORFs of LvReelers

Transcriptome data of *L. vannamei* stored in our laboratory were utilized to obtain cDNA fragments of *LvReelers*, followed by the design of suitable primers (Table 2) for amplifying the ORF sequences of *LvReeler1*, *LvReeler2*, and *LvReeler3*. The thermal cycling protocol comprised initial pre-denaturation (95 °C, 5 min); 30 cycles of sequential steps—DNA denaturation (95 °C, 30 s), primer annealing (58 °C, 30 s), and strand elongation (72 °C, 30 s); and terminal polymerization (72 °C, 10 min) to conclude. Amplified products underwent purification via the FastPure Gel DNA Extraction Mini Kit (Vazyme, Nanjing, China), followed by TA-cloning into pMD-19T vector (Takara, Kusatsu, Shiga, Japan). Recombinant plasmids were subsequently subjected to bidirectional sequencing through Tsingke Biotechnology Co., Ltd. (Beijing, China).

### 4.3. Bioinformatics Analysis

Nucleotide sequence homology searches and protein sequence retrieval across species were conducted using the BLAST 2.16.0 algorithm (http://www.ncbi.nlm.nih.gov/BLAST/, accessed on 15 December 2023). Physicochemical parameter predictions were facilitated through ExPASy (http://www.expasy.org, accessed on 17 October 2024), with functional domain characterization achieved via SMART (http://smart.emblheidelberg.de/, accessed on 20 December 2023). Sequence alignment procedures were performed with ClustalX 2.0 and GeneDoc 2.7 software packages. Phylogenetic reconstruction employing the NJ method was implemented in MEGA 6.0, incorporating bootstrap validation with 1000 replicate iterations.

### 4.4. Tissue Distribution and V. parahaemolyticus Challenge Assays

RNA isolation and cDNA synthesis were conducted using tissues (muscle, cardiac tissue, pereopods, pleopods, stomach, hemocytes, hepatopancreas, eyestalks, intestinal tissue, neural tissue, gill filaments, and epithelial layers) dissected from healthy *L. vannamei*. Following established protocols [36], a hemolymph was obtained via sterile pericardial puncture using a syringe preloaded with anticoagulant solution (0.34 M sodium chloride, 0.115 M glucose, 30 mM sodium citrate, and 10 mM EDTA). All other tissues were meticulously dissected under sterile conditions using surgical instruments and immediately flash-frozen in liquid nitrogen for subsequent processing. Primer pairs *qLvReeler*-F/R (Table 1) were employed for qRT-PCR quantification of *LvReeler* transcripts across tissue types. Reaction volumes of 10.0 μL were prepared, containing 5.0 μL 2× SYBR Green Master Mix (Takara, Kusatsu, Shiga, Japan), 1 μL cDNA template (1:10 diluted with ddH_2_O), and 250 nM primers. Amplification cycles were executed on a Bio-Rad CFX96 Real-Time PCR System under the following parameters: initial denaturation at 95 °C (3 min); 40 cycles of denaturation (95 °C, 5 s), annealing (60 °C, 30 s), and extension (78 °C, 5 s). Normalization utilized *EF-1α* (GenBank: GU136229) as the endogenous control, with primer sequences detailed in Table 1. Relative expression levels of *LvReelers* were calculated through the Livak (2^−ΔΔCt^) method [37], with technical triplicates implemented for all qRT-PCR analyses.

For immunological challenge studies, bacterial inoculum preparation followed established protocols [38]. One hundred twenty *L. vannamei* specimens were randomly allocated into four experimental cohorts (n = 30 per group). The treatment group received intramuscular injections of 50 μL *V. parahaemolyticus* suspension (2.18 × 10^6^ CFU/ mL) into the second and third abdominal somites, whereas PBS-injected controls were administered equivalent volumes. Tissue samples (hemocytes, hepatopancreas, and gills) were harvested at post-injection intervals (0, 4, 8, 12, 24, 48, and 72 h), with three biological replicates randomly collected per group at each timepoint. Transcript quantification of *LvReeler* was performed using the previously described qRT-PCR methodology.

### 4.5. RNA Interference and Survival Testing

dsRNA synthesis was performed in accordance with manufacturer protocols using the OneScribe T7 Transcription Kit (ABM, Vancouver, BC, Canada), incorporating methodologies from our prior study [39]. Nucleic acid integrity and concentration were verified through 1% agarose gel electrophoresis and SimpliNano spectrophotometry (GE Healthcare, Chicago, IL, USA). The synthesized dsRNA constructs demonstrated the following base pair lengths: *LvReelers* (401 bp, 387 bp, and 390 bp) and *GFP* (554 bp). Treatment cohorts received intramuscular delivery of *LvReelers* dsRNA (1 μg/g shrimp), while reference cohorts received equivalent doses of *GFP* dsRNA. Post-injection (48 h), gills from nine specimens per group were collected, with triplicate biological replicates generated by pooling every three shrimp samples. Subsequent RNA isolation and reverse transcription yielded cDNA templates for qRT-PCR quantification, utilizing *LvEF-1α* (primers in Table 1) as the endogenous normalization control.

Gill lesions are a typical symptom of *Vibrio* infection in shrimp, and the gills were selected to assess the bacterial load of *V. paraha*emolyticus. A cohort of 240 healthy *Litopenaeus vannamei* (6–8 g mean body weight, n = 60 per group) were allocated into four experimental cohorts. Intramuscular delivery of 50 μL PBS containing either *LvReelers* dsRNA or *GFP* dsRNA (1 μg/g shrimp) was administered to each cohort. Following a 48 h interval, secondary challenge with 50 μL *V. parahaemolyticus* suspension (2.18 × 10^6^ CFU/mL) was performed. Each cohort underwent bifurcation into two subcohorts: one designated for mortality monitoring at 4 h intervals (n = 30), and another for gills’ bacterial quantification. Survival analysis was conducted using the Mantel–Cox methodology (log-rank χ^2^ test). Furthermore, gill specimens collected 24 h post-infection were processed for *V. parahaemolyticus* load determination through established protocols [40].

### 4.6. Recombinant Expression and Purification of LvReelers

The ORFs of *LvReelers*, excluding signal peptide-encoding regions, were amplified via primers *pLvReeler*-R/F (Table 1) designed with protective nucleotides and restriction sites (*Kpn*I/*EcoR*I). Following purification, the *LvReeler* amplicons were ligated into the pET-32a expression vector and electroporated into *E. coli* Origami (DE3; Takara, Japan) for induction parameter optimization. Recombinant protein production was initiated under empirically determined conditions, followed by bacterial harvest and ultrasonic lysis. Clarified lysates underwent Ni-NTA affinity purification (Beyotime, Shanghai, China), subsequent dialysis, and concentration. Purity assessment was conducted through 15% SDS-PAGE, with quantitation performed using a BCA assay kit (Aidlab, Beijing, China). Parallel purification of rTrx served as negative control preparations.

### 4.7. SDS-PAGE and Western Blotting

Electrophoretic characterization of rLvReelers utilized standard 15% SDS-PAGE to assess protein expression profiles and purification efficiency. Sample preparation involved combining 1:1 (*v*/*v*) with loading buffer followed by thermal denaturation (100 °C, 5 min) prior to electrophoretic separation. Post-electrophoresis, gels underwent visualization via Coomassie Brilliant Blue R-250 (Beyotime, Shanghai, China) staining and subsequent destaining in methanol-acetic acid solution. For immunoblotting analyses, protein lysates were resolved via 15% SDS-PAGE followed by electrophoretic transfer to PVDF membranes. The expressions of rLvReelers were detected as described in our laboratory protocol [41], using 6× His-tagged rabbit monoclonal antibodies (Beyotime, Shanghai, China) as the primary antibodies.

### 4.8. Antibacterial Assay for rLvReelers

Three *Vibrio* species (*V. parahaemolyticus*, *V. alginolyticus*, and *V. harveyi*) were employed to evaluate antimicrobial efficacy of recombinant variants (rLvReeler1–3). MICs were determined by implementing established broth microdilution methodology [42,43]. Bacterial density was measured spectrophotometrically at 600 nm and standardized to an optical density (OD_600_) of 0.006. Sterile 96-well plates received 30 μL aliquots of either recombinant proteins (rLvReelers/rTRX-His-Tag) or PBS control, combined with 30 μL adjusted bacterial suspensions. Negative controls comprised 30 μL sterile PBS plus bacterial suspension. Following 24 h incubation at 28 °C, microbial proliferation was quantified via microplate spectrophotometry. Triplicate independent replicates ensured experimental reproducibility.

### 4.9. SEM Observation

Morphological alterations in *V. parahaemolyticus* and *V. alginolyticus* induced by rLvReeler3 were visualized via SEM. Bacterial suspensions were standardized to 1 × 10^8^ CFU/mL and combined with 80 μM rLvReeler3 at equal volumetric ratios. Following incubation at 28 °C for 1 h and 2 h, respectively, samples underwent overnight fixation at 4 °C with 2.5% glutaraldehyde, paralleled by rTRX-His-tag control treatments. Subsequent processing involved Freon-113 treatment (0.5%), sequential ethanol gradient dehydration, and critical point drying with CO_2_. Sputter-coated gold specimens were imaged using an Apreo field emission SEM (Thermo Fisher, Waltham, MA, USA).

### 4.10. Statistical Analysis

Data processing and statistical evaluations were conducted with GraphPad Prism 9.0 software. Intergroup mean differences were assessed through Student’s *t*-test for paired datasets. Statistical significance thresholds were defined as follows: probability values below 0.05 (*p* < 0.05) indicated significance, whereas *p*-values under 0.01 (*p* < 0.01) denoted strong statistical significance.

## 5. Conclusions

In this study, we successfully identified three single Reeler domain-containing proteins (rLvReeler1–3) from *L. vannamei* and preliminarily investigated their functions in antimicrobial immunity. The results demonstrated that the expression of *LvReelers* was upregulated upon *Vibrio* challenge, while gene silencing led to increased shrimp mortality and elevated bacterial load in gills. Furthermore, recombinant LvReeler proteins exhibited inhibitory effects against multiple *Vibrio* species, with rLvReeler3 showing the most vigorous activity. Mechanistically, rLvReeler3 likely exerts its antibacterial effect by disrupting bacterial aggregates, thereby interfering with normal physiological functions. Our findings enhance the understanding of the innate immune mechanisms in *L. vannamei* and provide novel insights and potential biocontrol strategies for *Vibrio* disease management in shrimp aquaculture. It is worth noting that it remains unclear whether LvReeler proteins are exclusively effective against *Vibrio* species or whether they also play a role in immune responses to other microorganisms. This question is crucial for a deeper understanding of the innate immune system of *L*. *vannamei*. Addressing this issue will contribute to the development of more targeted and effective disease control strategies for shrimp aquaculture. However, current research on LvReeler proteins remains incomplete. Future studies should address these limitations to elucidate their precise antimicrobial immune mechanisms, which would not only support the development of the aquaculture industry but also serve as a valuable reference for immunological research in other marine organisms, thereby advancing the field of marine immunology.

## Figures and Tables

**Table 1:** MIC of recombinant LvReeler proteins against *Vibrio*.

Microorganism (MIC, μM)	rLvReeler1 (MIC)	rLvReeler2 (MIC)	rLvReeler3 (MIC)
*Vibrio parahaemolyticus*	60	40	30
*Vibrio alginolyticus*	50	60	30
*Vibrio harveyi*	60	50	50

**Table 2:** PCR primers used in this study.

Primers	Primer Sequences (5′-3′)
For ORF cloning	
LvReeler1-F	GCTTTAGTGTCGTTACCCACCG
LvReeler1-R	TTAGGCCAACATCGCCTTAGCA
LvReeler2-F	CGACGAAGTGCGAGCGAGAA
LvReeler2-R	GGCGGAAGTGATGTGGAGTGAT
LvReeler3-F	CTGTTTTGTAAGAATGCTGCTGT
LvReeler3-R	TCTTAAACCTCTATGGGGTTGGA
For qPCR	
qLvReeler1-F	TGCCAAGGTGGTTGAGATGA
qLvReeler1-R	CCAACATCGCCTTAGCAGTC
qLvReeler2-F	CAGTCACCATCAAGGCTCAGAACAA
qLvReeler2-R	CGTGGCTGCTGTGTTGGGAAT
qLvReeler3-F	GGCACACAACAACGGCGATTT
qLvReeler3-R	GGATAACACGTCAGTCCAGTAGGT
qVP16s-F	GGTGTAGCGGTGAAATGCGTAG
qVP16s-R	CCACAACCTCCAAGTAGACATCG
For dsRNA Synthesis	
GFP-T7-F	GGATCCTAATACGACTCACTATAGGTCAGCGTGTCCGGCGAG
GFP-T7-R	GGATCCTAATACGACTCACTATAGGTCTTCTGCTTGTCGGC
GFP-F	TCAGCGTGTCCGGCGAG
GFP-R	TCTTCTGCTTGTCGGCC
LvReeler1-T7-F	GGATCCTAATACGACTCACTATAGGGTCTTTCACTTGCGGTTGCTG
LvReeler1-T7-R	GGATCCTAATACGACTCACTATAGGGAAGGTGACGATTCCAGCCA
LvReeler1-F	GTCTTTCACTTGCGGTTGCTG
LvReeler1-R	GAAGGTGACGATTCCAGCCA
LvReeler2-T7-F	GGATCCTAATACGACTCACTATAGGTGAGGTTGGTATTGGTAGGCG
LvReeler2-T7-R	GGATCCTAATACGACTCACTATAGGGGAAAACAGGGTTGGCGTCT
LvReeler2-F	TGAGGTTGGTATTGGTAGGCG
LvReeler2-R	GGAAAACAGGGTTGGCGTCT
LvReeler3-T7-F	GGATCCTAATACGACTCACTATAGGTGGGGTTTCAGCAGCGG
LvReeler3-T7-R	GGATCCTAATACGACTCACTATAGGGTCAGTCCAGTAGGTGCCGTAG
LvReeler3-F	TGGGGTTTCAGCAGCGG
LvReeler3-R	GTCAGTCCAGTAGGTGCCGTAG
For Protein Expression	
pLvReeler1-*Kpn*I-F	ggGGTACCATGCCCCTGCAAGTCACCCA
pLvReeler1-*EcoR*I-R	ggGAATTCTTAGGCCAACATCGCCTTAG
pLvReeler2-*Kpn*I-F	ggGGTACCTGGCCCAGCGGTGCCCCC
pLvReeler2-*EcoR*I-R	ggGAATTCCCTATATCATGAAGAACGAA
pLvReeler3-*Kpn*I-F	ggGGTACCTTCAGCAGCGGGAACGTGG
pLvReeler3-*EcoR*I-R	ggGAATTCTTAAACCTCTATGGGGTTGG

## Data Availability

The original data presented in the study are included in the article; further inquiries can be directed to the corresponding author.
